# Highly expressed proteins have an increased frequency of alanine in the second amino acid position

**DOI:** 10.1186/1471-2164-7-28

**Published:** 2006-02-16

**Authors:** Age Tats, Maido Remm, Tanel Tenson

**Affiliations:** 1 Department of Bioinformatics, Institute of Molecular and Cell Biology, University of Tartu, Riia 23, Tartu 51010, Estonia; 2 Institute of Technology, University of Tartu, Riia 23, Tartu 51010, Estonia

## Abstract

**Background:**

Although the sequence requirements for translation initiation regions have been frequently analysed, usually the highly expressed genes are not treated as a separate dataset.

**Results:**

To investigate this, we analysed the mRNA regions downstream of initiation codons in nine bacteria, three archaea and three unicellular eukaryotes, comparing the dataset of highly expressed genes to the dataset of all genes. In addition to the detailed analysis of the nucleotide and codon frequencies we compared the N-termini of highly expressed proteins to the N-termini of all proteins coded in the genome.

**Conclusion:**

The most conserved pattern was observed at the amino acid level: strong alanine over-representation was observed at the second amino acid position of highly expressed proteins. This pattern is well conserved in all three domains of life.

## Background

Initiation of translation is the basic determinant for the efficiency of translation. In bacteria the small ribosomal subunit, in complex with several initiation factors directly recognizes the translation initiation region (TIR) in mRNA. Determinants important for recognition of TIR are located between positions -20 and +15 [1], including mRNA secondary structure, purine-rich Shine-Dalgarno region (SD) (AGGAGG in *Escherichia coli*) [2-4], S1 protein binding A/U-rich enhancer [4-6], spacing between SD and start codon [7,8], the base immediately preceding the initiation codon [9] and the identity of the start codon [10]. These sequence motifs are directly involved in recruiting the initiating ribosomes. In addition, it has been found that codon usage at the beginning of open reading frames is non-random due to the selectional pressure for efficient gene expression [11,12], although precise nature of this pressure remains obscure. 15–20-fold effect on the levels of gene expression can be obtained by varying the codon following the initiation codon in the mRNA coding sequence; in *E. coli *AAA is the most common and most expression promoting codon in position +2 [13]. The overall preference for G-starting codons also positively correlated with gene expression level in *E. coli *[14]. On the other hand, NGG codons give strongly reduced gene expression [15]. The preference for A exists in about 20–30 nucleotide positions at the beginning of *E. coli *genes [16]. Suggestions that the downstream region influences translation initiation by mRNA-rRNA complementary base pairing failed to gain experimental support [17,18]. It has been shown that all single-stranded regions of 16S rRNAs have very high A content [19,20] despite of different genomic GC% [19]. Therefore it has been suggested that mRNA rich in A-residues is unstructured, thus being favourable for translation initiation [16,21,22].

In eukaryotes the small ribosomal subunit, in complex with several initiation factors and initiator tRNA, first recognizes the 5' end of mRNA and then scans to the initiation codon [23,24]. The efficiency of translation initiation is reduced if the sequence surrounding the AUG codon deviates significantly from certain preferred nucleotides. For example in *Saccharomyces cerevisiae *nucleotide context after initiation codon in highly expressed genes is shown to be AUGUC(U/C) [25-27].

The translation initiation mechanism of archaea is not clearly understood. Archaeal translation has both bacterial and eukaryotic characteristics [28-30]. Archaeal translation initiation factors are homologous to those of eukaryotes [31,32]. On the other hand, the calculations of the free energy values of the base-pairing between the 3' end of 16S rRNA and 5' UTR of mRNA in *Archaeoglobus fulgidus*, *Methanococcus jannaschii *and *Methanobacterium thermoautotrophicum *have shown a reduction in free-energy before the start codon; the patterns are similar to bacteria, but not to *Saccharomyces cerevisiae*, indicating the presence of a possible Shine-Dalgarno sequence in archaea [33]. Some archaea such as *Sulfolobus solfataricus *use two distinct mechanisms for translational initiation: SD-dependent initiation operates on distal cistrons of polycistronic mRNAs, whereas 'leaderless' initiation operates on monocistronic mRNAs and on opening cistrons of polycistronic mRNAs which start directly with the initiation codon [34].

Currently the genome sequences of many bacteria, archaea and eukaryotes are available. This provides a powerful tool for reconsidering the role of mRNA sequences in initiation of translation. As described above, there is evidence that the mRNA sequence immediately following the initiation codon can influence the efficiency of translation. We analysed the nucleotide preference downstream from the initiation codon in the genomes of 9 bacteria, 3 archaea and 3 unicellular eukaryotes. In addition to the detailed analysis of the nucleotide and codon frequencies we compared the N-termini of highly expressed proteins to the N-termini of all proteins coded in the genome. In contrast to many previous studies we have analysed the highly expressed genes as a separate dataset. This analysis identified sequence patterns in highly expressed genes, universal in all three domains of life.

## Results

### Adenosine frequencies at the beginning of *E. coli *ORFs

**Figure 1:**
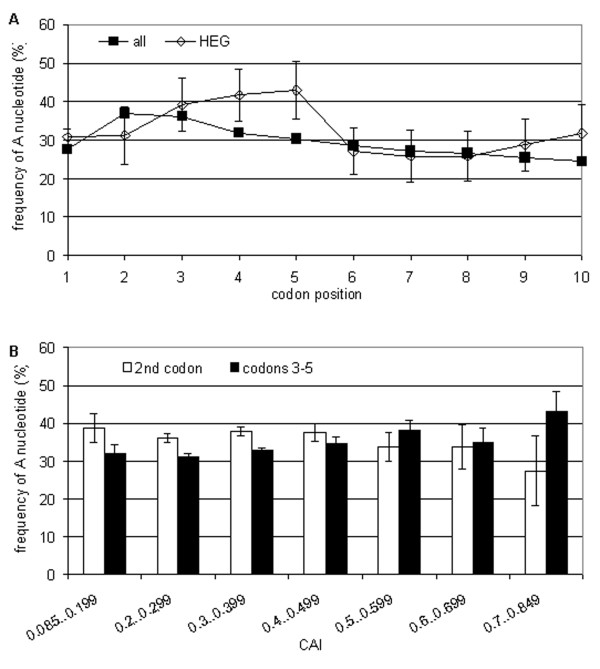
**Frequency of A at the beginning of *E. coli *ORFs**. Average frequency per codon is shown. **A**. Highly expressed genes have significant increase of A in codons 3–5 (nucleotides 7–15), but decrease in the second codon compared to the all genes dataset. **B**. The preference for A nucleotide in codon 2 (nucleotides 4–6) decreases with the increase of expression level. In contrast, there is a positive correlation between the expression level and the frequency of A in codons 3–5 (nucleotides 7–15). Error bars indicate 1.96 standard errors of the mean.

To study the sequence preferences at the beginnings of *Escherichia coli *ORFs we counted the nucleotide frequencies per codon as codon is functional unit in translation. In the first analysis we studied A content in all genes in the genome. Our results showed that the beginnings of ORFs had increased frequency of adenosine (A) (Fig. 1A). This is in agreement with previous observations that *E. coli *has a tendency towards A-rich sequences at the 3'-side of the initiation codon [16]. It has been suggested that this phenomenon is explained by the need to decrease the stability of mRNA secondary structure in the initiation site [13,16].

It is anticipated that the nucleotide preference pattern is even more pronounced in the most highly expressed genes. Codon adaptation index (CAI) characterizes how similar is synonymous codon usage in a given gene to the highly expressed genes. CAI values vary between 0 and 1. The CAI value of 1 is achieved when all amino acids in given gene are coded by the best codon in each synonymous codon family [35]. The correlation between codon adaptation index and expression level is well documented [36]. Therefore, A frequencies in 80 highly expressed genes (HEG) defined by the highest CAI value were analysed. It appeared that the frequency of A was 1.3 times higher in codons 3–5 of HEG comparing to dataset of all genes (P = 2.2E-06). In contrast, there was no increase in frequency of A nucleotide in the second codon. Rather, the frequency of A was decreased 1.3 times as compared to the dataset of all genes, although the statistical significance of the decrease was low (P = 0.079) (Fig. 1A).

To ensure that the difference in A nucleotide frequencies between codons 2 and 3–5 is related to the expression level of genes, the following analysis was performed: *E. coli *genes were subdivided into seven groups based on their CAI values and the A usage was compared in those groups. This analysis indicated that the preference of A nucleotide in 2 codon decreased only in the group of most highly expressed genes. In contrast, there was positive trend between CAI value and the frequency of A nucleotide in codons 3–5 (Fig. 1B).

### Nucleotide usage at the beginning of ORFs in different organisms

This pattern of the A nucleotide frequency can be specific to *E. coli *or it can be a more general phenomenon. In addition, the decrease of A in the second codon of HEG may be the result of regular changes in the frequencies of other nucleotides. To answer these questions, we analysed the nucleotide preference downstream from the initiation codon in the genomes of 9 bacteria, 3 archaea and 3 eukaryotes using the datasets of all genes and the most highly expressed genes.

Studied bacteria have a wide range of genome sizes, (the smallest is *M. genitalium *(0.5 Mbp) [37], the largest *E. coli *(4.6 Mbp) [38]), genomic GC%, (the lowest in *B. burgdorferi *(28.6 %) [39] and the highest in *M. tuberculosis *(65.6 %)[40]), different natural living environments (from parasites to free-living organisms) and different maximal growth-rates. The HEG datasets for bacteria other than *E. coli *were compiled based on the assumption that functional conservation implies conservation of relative gene expression level, a method successfully used in previous works (e.g. [41] and [42]). Accordingly, the HEG datasets consisted of orthologues to 80 HEG of *E. coli *(Additional file 1: Orthologues). The genomes of 3 archaea were also studied. The HEG datasets of archaea were compiled from orthologues to both 80 HEG of *E. coli *and 80 HEG of *S. cerevisiae *(Additional file 1: Orthologues). In addition to prokaryotes, we analysed the genomes of three eukaryotes. In multicellular organisms the codon usage pattern could be different in different tissues, possibly creating complexities that we could not treat in an appropriate manner. Therefore we confined our study with unicellular organisms, yeasts *S. cerevisiae *[43] and *S. pombe *[44] and the malaria parasite *P. falciparum *[45]. The HEG datasets of *S. pombe *and *P. falciparum *consisted of orthologues to 80 HEG of *S. cerevisiae *(Additional file 1: Orthologues).

**Figure 2:**
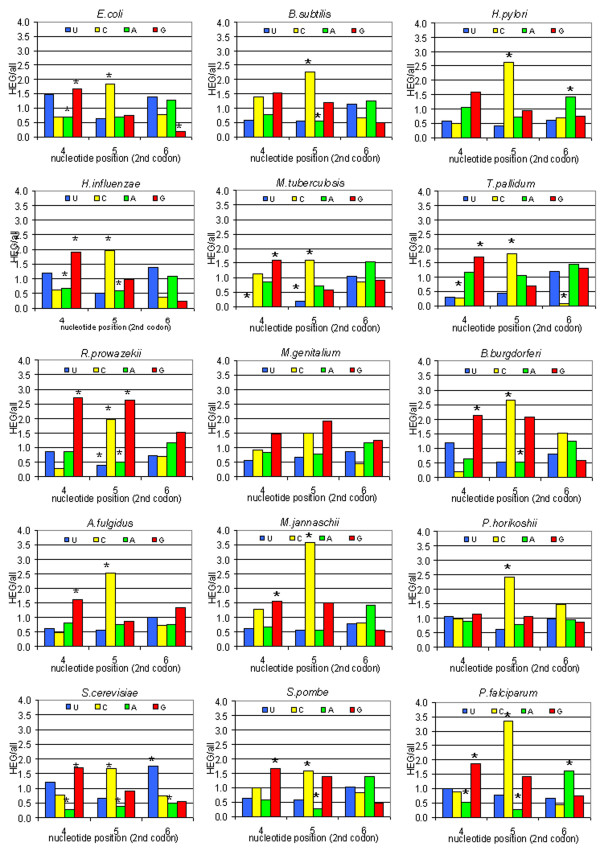
**Nucleotide usage in the second codon of HEG**. Nucleotide frequencies in all three positions of the second codon of HEG are divided by the corresponding frequencies of all genes. The asterisks mark significance probability less than 0.01 (H_0_: there is no difference of nucleotide frequencies between all genes and HEG).

Analysing the changes in nucleotide content of HEGs, we observed that the frequency of C in the fifth nucleotide of HEG (C_5_, corresponding to the second nucleotide of the second codon), was increased when compared to the all genes dataset (Fig. 2). In 14 of the 15 analysed genomes the increase was significant (P < 0.01). Only *M. genitalium *had no significant increase in the frequency of C_5_.

We also observed that the frequency of G in the fourth nucleotide of HEG (G_4_, corresponding to the first nucleotide of the second codon), tends to be increased in all studied genomes, although the increase is less noticeable. In 11 of the 15 genomes the increase was significant (P < 0.01). The increase of G_4 _and C_5 _was mostly accompanied with the decrease of A but in some cases also with the decrease in the frequencies of other nucleotides. (Fig. 2, Additional file 2: Pvalues).

**Figure 3:**
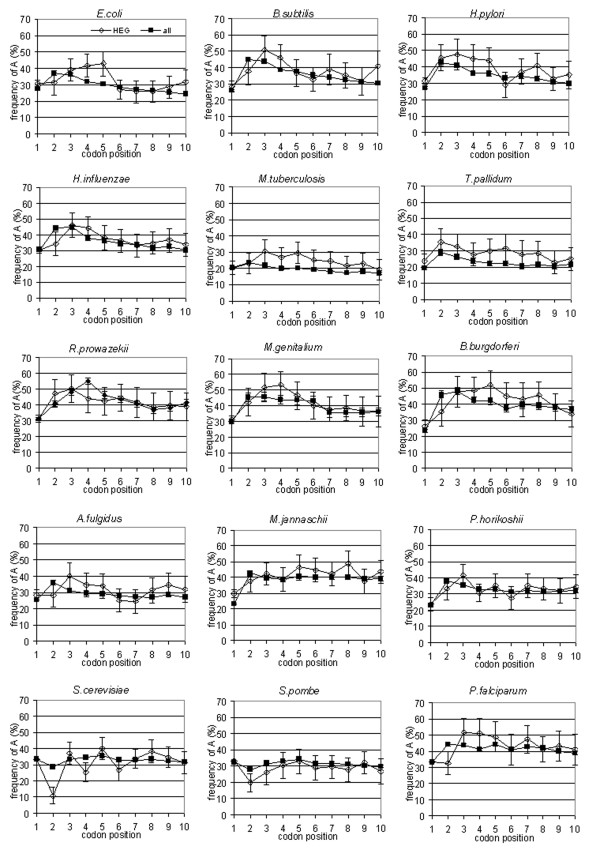
Frequency of A in the first 10 codons of HEG and in all genes.

In contrast to the increased frequency of G and C in the second codon, significant A increase in codons 3–5 (nucleotides 7–15) of HEG occurred in *M. tuberculosis *(P = 2.7E-06), in addition to *E. coli *(P = 2.2E-06) (Fig. 3). This phenomenon might be related to the need to decrease the stability of mRNA secondary structure in the initiation site [13,16]. Although this tendency is strong in *E. coli *(Fig. 1A) and *M. tuberculosis*, it is poorly conserved in other studied genomes (Fig. 3).

### Codon preferences

The observed nucleotide usage pattern suggests the preference for GCN as the second codon in HEG. Therefore, we compared the codon and amino acid usage at the beginnings of HEG with the beginnings of all genes (Table 1). Indeed, 11 of 15 organisms had significantly (P < 0.01) increased frequency of one of the GCN codons in the second codon. Sequence following the second codon (codons 3–5) had no common preference for certain codons in different organisms. None of the codons was significantly avoided at the beginning of HEG.

The increase in the frequency of GCN codons in the second codon position could be the result of the increased frequency of G_4 _and C_5 _(Additional file 2: Pvalues). In this case the overrepresentation of G_4 _and C_5 _would be independent of each other. Alternatively, the preference for GCN codons would create a nucleotide usage pattern where overrepresentation of G_4 _is correlated to the increased frequency of C_5_. To answer this question, we took out the genes with GNN codons in the second position from our analysis and tested for an increased frequency of C_5 _in the remaining datasets by comparing HEG to all genes in the genome. Similarly, we took out the genes with NCN codons in the second position and tested for an increased frequency of G_4 _in the remaining datasets. Slight overrepresentation of G_4 _and C_5 _was observed (Table 2) although this was much weaker than the over-representation of GCN codons in the second codon position of HEG (Table 1). The weak preference for other C_5_-codons or G_4_-codons apart from GCN codons can be also seen from the codon usage analysis (Table 1). Nevertheless, the preference for other codons is generally weaker than and not as conserved between different species as the preference for GCN codons.

In most cases, the usage of different GCN codons did not significantly differ between the second codon and the other positions in HEG (Table 3), indicating that there is a selection pressure to have the amino acid alanine, not any specific alanine codon at that position. In some genomes the frequency of GCA codons was significantly increased in the second position. This might relate to the increased frequency of A in codons 3–5 that is observed at least in some bacteria (Fig. 3).

### Amino acid preferences

In all studied organisms except *M. genitalium *the frequency of alanine as the second residue in highly expressed proteins was increased (Table 4). The overall preference for other amino acids in this position was also similar in different organisms: preferred amino acids in addition to alanine were glycine and serine. In positions 3, 4, 5 the amino acid preference pattern was not conserved. Still, in four genomes an increased frequency of positively charged amino acids was observed in at least one of these positions. This might be caused by the increased A frequency, as the lysine codons (AAG and AAA) and the overrepresented arginine codons (AGA) are A-rich. It is still interesting that codons AAG and AAA have been chosen from the set of all A-rich codons (Table 1). For example, the codons for asparagine (AAU and AAC) are not overrepresented in positions 3, 4 or 5.

## Discussion

We have found that HEG, when compared to the all genes dataset contain increased frequency of G nucleotide in the 4^th ^position and C in the 5^th ^position of the ORFs (Fig. 2). This tendency is correlated with the codon usage in the second position of HEG where the increased frequency of codons with G in the first and C in the second position is observed (Table 1). The amino acid usage pattern of the proteins coded by the HEG was even stronger: strong alanine (coded by the GCN codon family) overrepresentation was observed at second amino acid position of highly expressed proteins (Table 4). Moreover, the increased frequency of alanine is observed in all genomes analysed, except *M. genitalium *suggesting a universal feature for all highly expressed genes. Additional information about the selection of genomes for the study and finding the HEGs is presented in Additional files 3 and 4.

The influence of the nucleotides downstream from the initiation codon on the level of gene expression has been previously recognized both in bacteria and eukaryotes. On the other hand no general characteristic for HEG has been previously recognized. As the bacteria and eukaryotes use different mechanisms for initiation of translation, it has been anticipated that the initiation context effects are different. Our studies reveal a general pattern present in all three domains of life. What could be the reason for the observed nucleotide/amino acids usage pattern?

### Effects of the second codon

It has been previously shown that the second codon can influence the expression level of a gene. In *S. cerevisiae *the UCU codon is associated with increased expression level [26]. This is consistent with our observation that the frequency of UCU codon is increased in HEG although the increase of the GCN codons is most prominent (Table 1). In *E. coli *it has been shown that NGG codons cause low expression level [15]. This is reflected in the strongly decreased frequency of G as a third nucleotide of the second codon of HEG (Fig. 2). In *E. coli *the AAA codon has been associated with high expression level [13]. Therefore it is rather surprising that the frequency of AAA is not increased in the second codon position of HEG. Moreover, the frequency of A as a first nucleotide of the second codon is even decreased (Fig. 2). Similarly, the decreased frequency of A in the first or second position of the second codon is present in the HEGs of four other bacteria and all three eukaryotes analysed (Fig. 2).

One possible explanation might be related to the drop-off frequency of peptidyl-tRNA from the ribosome. It has been observed that during protein synthesis peptidyl-tRNA can sometimes dissociate from the ribosome instead of being separated into the protein and deaminoacylated tRNA in the termination reaction [46,47]. In case this drop-off reaction is very efficient, the enzyme responsible for recycling of peptidyl-tRNA, peptidyl-tRNA hydrolase, will be saturated. Therefore the tRNAs will accumulate in the peptidyl-tRNA form and the resulting shortage of deaminoacylated tRNA will not allow efficient translation [47-50]. The rate of this drop-off reaction depends on the length of the nascent peptide chain and on the codon. The shorter the peptide chain, the more efficient the drop-off is [49]. The peptidyl-tRNAs reading codons with A nucleotides in the first or second position are most prone to drop-off [51]. Therefore it is expected that A rich codons in the beginning of ORFs could cause high frequency of peptidyl-tRNA drop-off. As translation of the HEG provides most of the protein synthesis activity of the cell, the A rich codons might be avoided in the beginning of the ORFs to decrease the amount of drop-off products. Similarly, the GCN codons might be important for stabilizing the dipeptidyl-tRNA on the ribosome.

It is important to note that when the influence of the second codon on gene expression has been studied, the amount of the protein product has been measured. Another important parameter for understanding the role of different sequence elements might be the influence of protein overexpression on cell growth. In case some of the analysed sequences cause high level of peptidyl-tRNA drop-off, the growth would be inhibited. Even small inhibition of growth might be selected against at evolutionary scale and therefore influence the choice of sequences in HEG.

### Effects of the second amino acid

The first few N-terminal amino acid residues modulate the stability of proteins [52] and determine the cleavage of N-terminal formyl-methionine (or methionine in eukaryotes) [53-55]. It is possible that the observed nucleotide and codon preferences in highly expressed genes are caused by preference of these cleavage-promoting amino acids. The rules for formyl-methionine (or methionine) cleavage are similar in bacteria and eukaryotes [56,57]: the initiating amino acid is cleaved in case the second residue is alanine, glycine, proline, serine, threonine or valine. According to N-end rule, all those six amino acid residues are stabilizing in bacteria and also in *S. cerevisiae *[52]. Alanine, glycine and serine occurred as favourable amino acids in the second position, at least in some organisms (Table 4). We counted the number of proteins containing the Ala, Gly, Pro, Ser, Thr and Val residues in the second position of HEG and all genes datasets (Table 5). This analysis illustrates that the genes coding for proteins with cleavage determining and stabilizing residues in the second position are enriched within HEG. This is consistent with the high number of housekeeping genes in the HEG dataset. In case the observed sequence trends are caused by the selection for cleavage promoting and stabilizing amino acids in the beginning of HEG coded proteins, then it is interesting to note that alanine has been chosen from the set of six amino acids with similar properties. It is possible that the other amino acids are not as efficient as alanine in directing removal of the initiating amino acid and/or promoting protein stability.

In addition, it is also possible that alanine in the second position of the protein assists the entrance of the nascent peptide chain into the ribosomal tunnel [58]. In this context it is interesting to note that in addition to the increased frequency of alanine in the second position, the highly expressed proteins of several organisms contain positively charged amino acids in positions 3, 4 or 5 (Table 4). Therefore, N-termini of highly expressed proteins tend to have special characteristics that might influence their interaction with the ribosome.

## Conclusion

Strong alanine over-representation was observed at the second amino acid position of highly expressed proteins. This pattern is well conserved in all three domains of life.

## Methods

### Data

The protein coding sequences of following 9 bacteria, 3 archaea and 3 eukaryotes were retrieved from GenBank: *Escherichia coli K12 *[GenBank:NC_000913], *Bacillus subtilis *[GenBank:NC_000964], *Haemophilus influenzae *[GenBank:NC_000907], *Helicobacter pylori *26695 [GenBank:NC_000915], *Mycobacterium tuberculosis H37Rv *[GenBank:NC_000962], *Treponema pallidum *[GenBank:NC_000919], *Rickettsia prowazekii *[GenBank:NC_000963], *Mycoplasma genitalium *[GenBank:NC_000908], *Borrelia burgdorferi *[GenBank:NC_001318], *Methanococcus jannaschii *[GenBank:NC_000909], *Archaeoglobus fulgidus *[GenBank:NC_000917], *Pyrococcus horikoshii *[GenBank:NC_000961], *Saccharomyces cerevisiae *[GenBank:NC_001133-GenBank:NC_001148], *Schizosaccharomyces pombe *[GenBank:NC_003421, GenBank:NC_003423-GenBank:NC_003424], *Plasmodium falciparum *[GenBank:NC_000521, GenBank:NC_000910, GenBank:NC_004314-GenBank:NC_004318, GenBank:NC_004325-GenBank:NC_004331]. Two different datasets were compiled from each genome: one containing highly expressed genes (HEG) and the other set consisting of the all genes of the corresponding organism.

### Highly expressed genes

For dataset of 80 HEG of *E. coli *and *S. cerevisiae *we chose 80 genes having the highest codon adaptation index (CAI) [35], which was calculated by using program CodonW [59]. Calculation of CAI is based on a dataset of highly expressed genes including genes coding ribosomal proteins, outer membrane proteins, elongation factors, heat shock proteins and RNA polymerase subunits [35]. The HEG datasets for the rest of the studied organisms were compiled based on the assumption that functional conservation implies the conservation of relative gene expression level, method successfully used in previous works (for example [41] and [42]). Therefore, the HEG dataset for the rest of the studied bacteria consisted of orthologues to those 80 HEG of *E. coli*; the HEG datasets of *S. pombe *and *P. falciparum *consisted of orthologues to 80 HEG of *S. cerevisiae *and the HEG datasets of archaea were compiled from orthologues to both 80 HEG of *E. coli *and 80 HEG of *S. cerevisiae *(Additional file 1: Orthologues). Orthologues were found by comparing two genomes with reciprocal BLAST search [60] and selecting mutually best hits by using the program INPARANOID [61].

### Statistical significance

We used two-tailed Fisher's exact test (FET) to compare observed frequencies of nucleotide, codon or amino acid in HEG dataset and all genes dataset (all genes dataset contains HEG as a subset). FET examines whether the frequencies in two datasets are different enough to reject the null hypothesis (the exact meanings of null hypothesis for each analysis are described in figure legends). In all figures and tables the P-values of 0.01 or less were considered significant. No correction for multiple testing was applied to any of the analyses.

## Authors' contributions

AT performed the data analysis. MR participated in the study's design and choice of methods. TT conceived the study and participated in its design and coordination. All authors read and approved the final manuscript.

## Supplementary Material

Additional File 1 The list of orthologues to 80 HEG of *E. coli *and to 80 HEG of *S. cerevisiae*. Click here for file

Additional File 2 P-values for U, C, A and G in nucleotide positions 4–30. H_0_: there is no difference in nucleotide frequency between all genes and HEG. (ecoli – *E. coli*, bsubt – *B. subtilis*, hpylo – *H. pylori*, hinfl – *H. influenzae*, mtube – *M. tuberculosis*, tpall – *T. pallidum*, rprow – *R. prowazekii*, mgeni – *M. genitalium*, bburg – *B. burgdorferi*, afulg – *A. fulgidus*, mjann – *M. jannaschii*, phori – *P. horikoshii*, scere – *S. cerevisiae*, spomb – *S. pombe*, pfalc – *P. falciparum*). ↑: frequency is increased in HEG compared to all genes. ↓: frequency is decreased in HEG compared to all genes. -: no difference between HEG and all genes datasets. Click here for file

Additional File 3 Justifying the method of using orthologues. Click here for file

Additional File 4 Justifying the selection of organism. Click here for file

## Figures and Tables

**Table 1:** Preference for codons at the beginning of HEG compared to all genes datasets. (H_0_: there is no difference between codon frequencies in all genes and HEG).

organism	codon position											
	
	2			3			4			5		
	
	codon	P-value	%HEG/ %all	codon	P-value	%HEG/ %all	codon	P-value	%HEG/ %all	codon	P-value	%HEG/ %all
*E. coli*	**GCU**	7.9E-06	15/3	ACU	1.1E-04	10/2	AUU	4.0E-05	16/4	AAA	0.001	15/5
	**GCA**	6.0E-04	11/3	AAG	0.004	11/4	ACU	0.007	6/1			
	UCC	7.1E-04	8/1									
*B. subtilis*	**GCA**	8.5E-05	15/3	GUA	0.004	9/2	GGA	0.002	9/2		-	
*H. pylori*	**GCA**	0.001	15/4		-			-		AAG	0.009	9/2
	ACA	0.005	6/1									
*H. influenzae*	**GCA**	1.0E-04	17/4	GUA	0.005	6/1	GAA	0.009	11/4		-	
	**GCU**	0.002	11/3									
	UCU	0.006	10/3									
*M. tuberculosis*	**GCA**	3.1E-04	11/2	AAG	2.9E-04	11/2	AAG	2.1E-04	11/2	AAG	8.5E-05	11/2
	**GCG**	0.008	11/4							ACU	0.008	5/1
*T. pallidum*	**GCA**	6.9E-04	15/3	AAG	0.010	13/4		-			-	
*R. prowazekii*		-			-			-			-	
*M. genitalium*		-			-			-			-	
*B. burgdorferi*	**GCA**	1.0E-04	15/2		-			-			-	
	UCA	9.9E-04	10/1									
	GGU	0.004	8/1									
*A. fulgidus*		-			-		AAG	0.004	15/5	AAG	0.002	15/4
										UAU	0.006	8/1
										AGA	0.006	11/3
*M. jannaschii*	**GCA**	1.1E-05	14/2	GGA	0.003	9/2		-			-	
	**GCU**	0.002	9/2									
	CCA	0.008	7/1									
*P. horikoshii*		-			-		AUG	0.004	27/12		-	
*S. cerevisiae*	**GCU**	7.3E-08	19/3	AGA	2.5E-07	18/3	GUU	8.6E-08	15/2	AAG	8.4E-04	13/4
	UCU	7.2E-07	24/6	CCA	0.006	6/1	GGU	0.002	8/2	ACU	0.001	11/3
	GGU	1.0E-05	13/2				CCA	0.005	8/2	GUU	0.002	9/2
	**GCC**	0.006	6/1									
*S. pombe*	**GCA**	2.0E-07	22/4	CGU	4.5E-06	13/1		-		AAG	8.7E-04	13/3
	GGA	0.002	8/1	AUU	0.001	13/3				AUC	0.009	7/1
*P. falciparum*	GGA	0.001	11/2		-		CAA	0.004	11/2		-	
	**GCA**	0.001	11/2									
	UCA	0.002	11/2									
	**GCU**	0.002	11/2									
	CCA	0.006	6/1									

**Table 2:** Ratio of G_4 _and C_5 _nucleotides between HEG and all genes datasets. For test of G_4_, genes with NCN codons and for test of C_5_, genes with GNN codons in the second position were removed from datasets. (H_0_: there is no difference between nucleotide frequencies in all genes and HEG).

organism	G_4_		C_5_	
		
	P-value	HEG/all ratio	P-value	HEG/all ratio
*E. coli*	1.000	1.0	0.048	1.5
*B. subtilis*	0.490	0.7	0.111	1.7
*H. pylori*	0.644	1.2	0.004	2.6
*H. influenzae*	0.336	1.4	0.010	1.8
*M. tuberculosis*	0.010	0.0	0.495	1.2
*T. pallidum*	0.524	1.2	1.000	0.9
*R. prowazekii*	0.012	2.8	0.010	1.9
*M. genitalium*	0.298	1.3	0.763	1.2
*B. burgdorferi*	0.011	2.1	0.011	2.6
*A. fulgidus*	0.177	1.4	0.073	2.0
*M. jannaschii*	0.282	1.3	0.033	2.5
*P. horikoshii*	1.000	1.0	0.030	2.2
*S. cerevisiae*	0.006	1.8	2.8E-04	1.7
*S. pombe*	0.011	1.7	0.010	1.6
*P. falciparum*	0.004	2.1	1.3E-05	3.9

**Table 3:** Frequency of GCN codons in the second position of HEG and in entire HEG of different organisms. (H_0_: there is no difference between GCN codon frequencies in the second codon of HEG and in all codons of HEG).

		percentage of codon					percentage of codon		
							
organism	codon	2nd Position of HEG	entire HEG	P-value	organism	codon	2nd Position of HEG	entire HEG	P-value
*E. coli*	GCU	57.1	41.8	0.184	*A. fulgidus*	GCU	21.4	24.4	1.000
	GCC	0.0	8.8	0.250		GCC	21.4	22.7	1.000
	GCA	42.9	25.7	0.082		GCA	28.6	28.0	1.000
	GCG	0.0	23.7	**0.007**		GCG	28.6	24.8	0.758

*B. subtilis*	GCU	33.3	40.2	0.792	*M. jannaschii*	GCU	38.5	48.3	0.582
	GCC	0.0	8.1	0.625		GCC	0.0	4.3	1.000
	GCA	53.3	31.3	0.091		GCA	61.5	44.7	0.268
	GCG	13.3	20.4	0.749		GCG	0.0	2.8	1.000

*H. pylori*	GCU	28.6	41.3	0.419	*P. horikoshii*	GCU	0.0	38.0	0.049
	GCC	0.0	16.6	0.144		GCC	42.9	22.6	0.199
	GCA	57.1	11.8	**7.3E-05**		GCA	28.6	31.0	1.000
	GCG	14.3	30.2	0.251		GCG	28.6	8.4	0.113

*H. influenzae*	GCU	40.0	26.1	0.199	*S. cerevisiae*	GCU	68.2	75.7	0.453
	GCC	0.0	4.9	0.621		GCC	22.7	23.4	1.000
	GCA	60.0	50.4	0.502		GCA	9.1	0.6	**0.010**
	GCG	0.0	18.7	0.037		GCG	0.0	0.3	1.000

*M. tuberculosis*	GCU	19.2	8.0	0.054	*S. pombe*	GCU	17.6	62.5	**2.2E-04**
	GCC	26.9	49.4	0.029		GCC	5.9	31.5	0.031
	GCA	26.9	7.6	**0.003**		GCA	76.5	5.3	**1.4E-13**
	GCG	26.9	35.0	0.535		GCG	0.0	0.7	1.000

*T. pallidum*	GCU	21.4	20.3	1.000	*P. falciparum*	GCU	45.5	53.6	0.763
	GCC	0.0	12.2	0.397		GCC	0.0	14.8	0.382
	GCA	50.0	24.7	0.055		GCA	45.5	31.1	0.334
	GCG	28.6	42.8	0.416		GCG	9.1	0.4	0.061

*R. prowazekii*	GCU	44.4	45.4	1.000					
	GCC	11.1	4.8	0.365					
	GCA	44.4	42.2	1.000					
	GCG	0.0	7.6	1.000					

*M. genitalium*	GCU	33.3	48.9	0.507					
	GCC	0.0	7.2	1.000					
	GCA	66.7	39.8	0.168					
	GCG	0.0	4.1	1.000					

*B. burgdorferi*	GCU	22.2	50.3	0.177					
	GCC	0.0	8.0	1.000					
	GCA	77.8	36.5	0.015					
	GCG	0.0	5.2	1.000					

**Table 4:** Preference for amino acids at the beginning of highly expressed proteins compared to all proteins datasets. (H_0_: there is no difference of amino acid frequencies between all proteins and highly expressed proteins).

organism	amino acid position											
	
	2			3			4			5		
	
	amino acid	P-value	%HEG/%all	amino acid	P-value	%HEG/%all	amino acid	P-value	%HEG/%all	amino acid	P-value	%HEG/%all
*E. coli*	**Ala**	3.5E-06	26/9	Lys	0.004	23/11	Ile	7.2E-04	23/9	Lys	3.8E-05	21/7

*B. subtilis*	**Ala**	3.6E-06	28/7		-		Gly	0.003	13/3		-	

*H. pylori*	**Ala**	1.0E-04	26/8		-			-			-	

*H. influenzae*	**Ala**	3.0E-06	29/9		-			-		Val	0.008	13/5
	Ser	0.007	23/11									

*M. tuberculosis*	**Ala**	6.4E-09	43/12	Lys	1.3E-04	15/3	Tyr	0.005	8/2	Lys	2.5E-04	13/3
							Lys	0.003	11/3	Thr	0.007	16/6

*T. pallidum*	**Ala**	3.7E-05	29/8		-			-			-	

*R. prowazekii*	**Ala**	7.6E-04	18/5	Thr	0.003	18/5		-			-	
	Ser	0.007	27/11									

*M. genitalium*		-			-			-			-	

*B. burgdorferi*	**Ala**	3.4E-04	19/4		-			-			-	
	Gly	3.4E-04	15/3									

*A. fulgidus*	**Ala**	8.6E-06	26/7		-			-			-	

*M. jannaschii*	**Ala**	1.8E-07	23/4		-		Arg	0.009	13/4		-	

*P. horikoshii*	**Ala**	0.007	13/4		-		Met	0.004	27/12		-	

*S. cerevisiae*	**Ala**	3.5E-07	28/8	Arg	1.0E-05	21/6	Val	1.0E-04	18/5	Lys	0.002	20/9
	Gly	0.002	14/5							Leu	0.002	0/9

*S. pombe*	**Ala**	6.4E-05	28/10		-			-			-	
	Gly	6.3E-04	17/5									

*P. falciparum*	**Ala**	1.2E-05	23/5		-		Gln	0.008	11/3		-	
	Asn	0.003	0/12									

**Table 5:** The percentage of proteins containing the Ala, Gly, Pro, Ser, Thr and Val residues in the second position of highly expressed proteins and all proteins. (H_0_: there is no difference in frequency of this group of amino acids in the second position of highly expressed proteins and in the second position of all proteins).

organism	Percentage of Ala, Ser, Thr, Gly, Val and Pro in the 2nd position		P-value
		
	HEG	all	
*E. coli*	60	40	4.8E-04
*B. subtilis*	58	32	8.9E-05
*H. pylori*	56	28	4.1E-05
*H. influenzae*	64	36	3.2E-06
*M. tuberculosis*	80	63	0.005
*T. pallidum*	52	38	0.068
*R. prowazekii*	71	31	2.8E-08
*M. genitalium*	44	29	0.040
*B. burgdorferi*	58	24	1.3E-06
*A. fulgidus*	55	30	2.0E-04
*M. jannaschii*	54	26	2.0E-05
*P. horikoshii*	48	31	0.015
*S. cerevisiae*	85	54	1.0E-08
*S. pombe*	90	52	6.6E-10
*P. falciparum*	79	28	1.5E-12
