# Supplementary material for: Highly expressed proteins have an increased frequency of alanine in the second amino acid position
Source: BMC Genomics. 2006 Feb 16;7:28. doi: 10.1186/1471-2164-7-28 (PMC1397820; doi:10.1186/1471-2164-7-28)
Supplement: Additional File 2 — P-values for U, C, A and G in nucleotide positions 4–30. H0: there is no difference in nucleotide frequency between all genes and HEG. (ecoli – E. coli, bsubt – B. subtilis, hpylo – H. pylori, hinfl – H. influenzae, mtube – M. tuberculosis, tpall – T. pallidum, rprow – R. prowazekii, mgeni – M. genitalium, bburg – B. burgdorferi, afulg – A. fulgidus, mjann – M. jannaschii, phori – P. horikoshii, scere – S. cerevisiae, spomb – S. pombe, pfalc – P. falciparum). ↑: frequency is increased in HEG compared to all genes. ↓: frequency is decreased in HEG compared to all genes. -: no difference between HEG and all genes datasets. [file 1471-2164-7-28-S2.pdf]

U nucleotide ↑: frequency is increased in HEG compared to all genes.↓: frequency is decreased in HEG compared to all genes

nucleotide	organism														
position	ecoli	bsubt	hpylo	hinfl	mtube	tpall	rprow	mgeni	bburg	afulg	mjann	phori	scere	spomb	pfalc
4	0.108 ↑	0.306 ↓	0.301 ↓	0.513 ↑	**0.001** ↓	0.052 ↓	0.851 ↓	0.340 ↓	0.703 ↑	0.477 ↓	0.396 ↓	0.808 ↑	0.305 ↑	0.127 ↓	1.000 ↓
5	0.099 ↓	0.122 ↓	0.011 ↓	0.037 ↓	**0.003** ↓	0.084 ↓	**0.007** ↓	0.350 ↓	0.036 ↓	0.059 ↓	0.016 ↓	0.068 ↓	0.128 ↓	0.168 ↓	0.482 ↓
6	0.037 ↑	0.545 ↑	0.070 ↓	0.036 ↑	0.865 ↑	0.414 ↑	0.219 ↓	0.621 ↓	0.361 ↓	1.000 ↓	0.372 ↓	1.000 ↓	**1.0E-06** ↑	0.794 ↑	0.070 ↓
7	0.031 ↓	0.564 ↓	0.046 ↓	0.051 ↓	0.096 ↓	0.162 ↓	0.414 ↓	0.308 ↓	0.710 ↓	0.849 ↑	0.865 ↓	0.073 ↓	0.181 ↓	0.293 ↓	0.141 ↓
8	0.705 ↓	0.214 ↑	0.656 ↓	0.691 ↓	0.653 ↑	0.614 ↑	0.337 ↑	0.228 ↓	0.875 ↑	0.305 ↓	0.037 ↓	0.302 ↓	0.440 ↓	1.000 ↓	**0.005** ↓
9	0.024 ↑	0.157 ↓	0.771 ↓	0.364 ↓	0.322 ↓	0.504 ↑	0.363 ↓	0.190 ↓	0.648 ↓	0.046 ↓	0.155 ↓	**0.007** ↓	0.050 ↓	**0.014** ↑	1.000 ↓
10	0.036 ↓	**0.008** ↓	**0.007** ↓	0.260 ↓	0.183 ↑	0.851 ↓	0.243 ↓	0.404 ↓	0.110 ↓	0.012 ↓	0.610 ↓	0.848 ↑	0.895 ↓	0.282 ↓	0.401 ↓
11	0.555 ↑	1.000 ↓	1.000 ↑	0.529 ↓	0.768 ↓	0.336 ↑	0.179 ↓	0.087 ↓	0.545 ↓	0.397 ↓	0.021 ↓	0.066 ↑	0.031 ↑	0.666 ↓	1.000 ↓
12	**1.0E-03** ↑	0.648 ↑	0.771 ↓	0.258 ↑	0.733 ↓	0.871 ↑	1.000 ↓	0.022 ↓	0.444 ↓	0.410 ↑	0.142 ↓	1.000 ↑	**0.007** ↑	0.346 ↓	0.073 ↓
13	1.000 ↓	0.488 ↓	0.072 ↓	0.042 ↓	0.201 ↓	0.364 ↓	0.394 ↓	0.446 ↑	0.064 ↓	0.851 ↑	0.276 ↓	0.123 ↓	**0.001** ↓	0.019 ↓	0.238 ↓
14	0.407 ↑	0.559 ↑	1.000 ↑	0.315 ↑	0.314 ↓	0.267 ↑	0.880 ↓	0.409 ↓	0.230 ↓	0.022 ↓	0.098 ↓	**0.008** ↓	0.524 ↓	0.550 ↑	0.632 ↑
15	0.180 ↓	0.368 ↑	0.079 ↓	0.134 ↑	0.861 ↑	0.109 ↑	0.378 ↑	1.000 ↓	1.000 ↑	0.754 ↑	0.663 ↓	0.634 ↑	0.335 ↑	0.417 ↑	0.100 ↓
16	0.020 ↓	0.865 ↓	0.875 ↓	0.031 ↓	0.457 ↓	0.380 ↓	0.732 ↑	0.170 ↓	0.519 ↓	0.012 ↓	0.869 ↓	1.000 ↓	1.000 ↓	0.097 ↓	0.183 ↓
17	0.906 ↓	0.887 ↑	0.393 ↓	0.377 ↓	0.239 ↑	0.426 ↓	0.881 ↑	0.011 ↑	0.097 ↓	0.574 ↓	0.073 ↓	0.114 ↓	0.710 ↓	0.772 ↓	0.638 ↓
18	0.144 ↑	0.167 ↑	0.668 ↑	0.706 ↑	0.363 ↓	0.530 ↑	0.762 ↓	0.628 ↑	0.368 ↓	0.528 ↑	1.000 ↑	**0.007** ↑	**6.0E-05** ↑	0.417 ↓	1.000 ↓
19	0.191 ↓	0.730 ↓	0.430 ↓	0.149 ↓	0.432 ↓	0.857 ↓	0.729 ↓	0.579 ↑	0.230 ↓	0.465 ↑	0.177 ↓	0.064 ↓	0.895 ↑	0.073 ↓	0.014 ↓
20	1.000 ↑	0.112 ↓	1.000 ↑	0.533 ↓	0.186 ↑	0.535 ↓	0.286 ↓	0.425 ↑	0.010 ↓	0.395 ↓	0.217 ↓	0.083 ↓	0.110 ↓	0.399 ↓	0.012 ↓
21	1.000 ↑	0.544 ↓	0.382 ↓	0.619 ↑	0.150 ↓	0.422 ↑	0.181 ↑	0.430 ↓	0.650 ↓	0.527 ↑	0.379 ↓	0.220 ↑	**0.008** ↓	1.000 ↑	0.655 ↑
22	0.312 ↓	0.610 ↓	0.011 ↓	1.000 ↓	1.000 ↓	0.059 ↓	**0.008** ↓	0.268 ↓	0.010 ↓	0.192 ↓	0.488 ↓	0.578 ↓	**0.009** ↓	0.022 ↓	0.061 ↓
23	0.406 ↑	0.391 ↓	0.254 ↓	0.800 ↑	1.000 ↓	0.426 ↓	**0.004** ↓	0.332 ↓	**5.1E-05** ↓	0.312 ↑	0.091 ↓	0.151 ↓	0.017 ↓	0.392 ↓	0.644 ↓
24	0.805 ↓	0.175 ↑	0.660 ↓	0.618 ↓	0.352 ↑	0.520 ↑	0.883 ↑	0.637 ↓	1.000 ↑	1.000 ↓	0.309 ↓	0.121 ↑	0.116 ↑	0.181 ↑	0.881 ↑
25	**0.002** ↓	1.000 ↓	0.349 ↓	0.037 ↓	0.079 ↓	0.084 ↓	0.163 ↓	0.575 ↓	0.141 ↓	0.194 ↓	0.328 ↑	0.274 ↓	0.414 ↓	0.059 ↓	0.021 ↓
26	0.811 ↓	0.772 ↓	0.476 ↓	1.000 ↓	0.455 ↑	0.521 ↓	0.294 ↓	0.748 ↑	1.000 ↓	0.243 ↓	0.671 ↓	**0.002** ↓	0.207 ↑	0.322 ↓	0.286 ↓
27	0.383 ↓	0.881 ↓	0.105 ↓	0.311 ↓	1.000 ↓	0.520 ↑	0.459 ↑	1.000 ↓	0.293 ↓	0.408 ↓	0.475 ↓	0.438 ↓	0.469 ↓	0.059 ↓	0.369 ↑
28	0.100 ↓	0.215 ↓	0.154 ↓	0.017 ↓	0.841 ↓	0.459 ↓	0.489 ↓	0.560 ↓	0.045 ↓	0.144 ↓	0.408 ↓	0.143 ↓	0.137 ↓	**1.1E-04** ↓	**0.001** ↓
29	0.230 ↓	0.060 ↓	0.393 ↓	0.094 ↓	0.392 ↓	0.632 ↑	0.882 ↑	0.513 ↓	0.458 ↓	0.026 ↓	0.067 ↓	0.559 ↓	0.617 ↓	0.118 ↓	0.092 ↓
30	0.298 ↓	1.000 ↑	0.061 ↓	0.531 ↑	0.711 ↓	0.310 ↑	1.000 ↓	0.266 ↑	0.548 ↑	0.747 ↑	0.773 ↓	0.759 ↑	0.148 ↑	0.418 ↑	0.298 ↓

C nucleotide ↑: frequency is increased in HEG compared to all genes.↓: frequency is decreased in HEG compared to all genes

nucleotide position	organism														
	ecoli	bsubt	hpylo	hinfl	mtube	tpall	rprow	mgeni	bburg	afulg	mjann	phori	scere	spomb	pfalc
4	0.240 ↓	0.272 ↑	0.064 ↓	0.185 ↓	0.632 ↑	**0.006** ↓	0.051 ↓	1.000 ↓	0.079 ↓	0.125 ↓	0.465 ↑	1.000 ↓	0.506 ↓	1.000 ↓	0.506 ↓
5	**2.2E-05** ↑	**2.1E-05** ↑	**3.4E-06** ↑	**5.0E-06** ↑	**2.6E-05** ↑	**0.010** ↑	**0.001** ↑	0.123 ↑	**1.5E-04** ↑	**3.0E-05** ↑	**8.7E-07** ↑	**6.3E-04** ↑	**6.3E-06** ↑	**0.001** ↑	**6.3E-06** ↑
6	0.392 ↓	0.428 ↓	0.510 ↓	0.055 ↓	0.358 ↓	**3.8E-05** ↓	0.792 ↓	0.416 ↓	0.375 ↑	0.470 ↓	1.000 ↓	0.163 ↑	0.313 ↓	0.711 ↓	0.313 ↓
7	0.673 ↓	0.582 ↓	0.134 ↑	0.033 ↑	0.879 ↑	1.000 ↓	0.530 ↓	0.820 ↓	0.762 ↑	1.000 ↓	0.179 ↓	0.040 ↑	0.876 ↓	0.073 ↑	0.876 ↑
8	1.000 ↑	0.256 ↓	1.000 ↓	0.201 ↑	0.211 ↓	0.050 ↓	0.120 ↑	0.379 ↑	0.781 ↑	0.698 ↑	0.052 ↑	0.838 ↓	0.363 ↓	0.882 ↑	0.363 ↑
9	**0.004** ↓	1.000 ↓	1.000 ↑	0.306 ↓	0.503 ↑	0.151 ↓	1.000 ↓	1.000 ↓	0.355 ↑	0.089 ↓	0.380 ↑	0.080 ↑	0.025 ↑	0.477 ↓	0.025 ↓
10	0.030 ↓	0.214 ↓	0.829 ↓	0.038 ↓	0.119 ↓	0.861 ↓	0.824 ↓	0.673 ↑	0.791 ↓	0.331 ↓	0.285 ↑	0.672 ↓	0.143 ↓	0.395 ↑	0.143 ↑
11	0.680 ↓	0.854 ↑	0.854 ↓	0.751 ↓	0.784 ↑	**0.730** ↓	0.387 ↓	0.525 ↑	0.814 ↑	0.089 ↓	0.119 ↑	0.673 ↓	0.694 ↑	0.767 ↓	0.694 ↓
12	0.413 ↓	0.075 ↓	0.373 ↓	0.363 ↓	0.105 ↑	0.722 ↓	0.793 ↓	0.817 ↑	0.772 ↑	0.331 ↓	0.321 ↑	0.228 ↓	0.380 ↑	**0.004** ↑	0.380 ↓
13	0.014 ↓	0.214 ↓	0.168 ↓	0.434 ↓	**0.001** ↓	0.090 ↓	1.000 ↓	0.820 ↓	0.245 ↓	0.331 ↓	0.314 ↓	0.309 ↑	0.561 ↑	0.742 ↓	0.561 ↓
14	0.040 ↓	0.576 ↑	1.000 ↓	0.649 ↓	0.395 ↑	1.000 ↓	0.842 ↑	0.824 ↑	0.599 ↑	0.052 ↓	0.823 ↑	0.439 ↑	0.235 ↑	0.088 ↑	0.235 ↑
15	0.688 ↑	0.170 ↑	1.000 ↓	0.868 ↑	0.790 ↑	0.090 ↓	0.793 ↓	0.101 ↓	0.355 ↓	0.039 ↓	0.800 ↑	0.705 ↓	0.549 ↓	0.016 ↑	0.549 ↓
16	0.606 ↑	0.711 ↑	0.701 ↑	0.236 ↑	0.884 ↑	1.000 ↑	1.000 ↓	0.823 ↑	0.201 ↑	1.000 ↓	1.000 ↓	0.319 ↑	0.367 ↓	0.863 ↓	0.367 ↑
17	0.500 ↓	0.026 ↓	0.848 ↑	1.000 ↓	0.124 ↓	0.098 ↓	0.670 ↑	0.636 ↓	0.644 ↓	0.269 ↑	0.043 ↑	0.438 ↑	0.092 ↑	0.355 ↑	0.092 ↑
18	0.344 ↓	0.258 ↓	0.373 ↓	0.617 ↓	0.691 ↑	1.000 ↓	0.570 ↓	0.809 ↑	0.573 ↓	0.420 ↓	0.637 ↑	0.717 ↓	0.652 ↑	**0.004** ↑	0.652 ↓
19	0.237 ↑	0.098 ↑	0.690 ↑	1.000 ↓	0.569 ↓	0.730 ↓	0.672 ↑	0.432 ↑	0.795 ↑	0.854 ↓	0.087 ↑	0.697 ↑	0.300 ↓	0.226 ↑	0.300 ↑
20	1.000 ↓	0.582 ↓	0.453 ↑	0.341 ↑	0.492 ↓	0.217 ↓	1.000 ↓	0.832 ↓	0.486 ↑	0.263 ↑	0.051 ↑	0.564 ↑	0.138 ↑	0.878 ↓	0.138 ↓
21	0.795 ↑	0.597 ↑	0.585 ↑	0.402 ↓	0.235 ↑	0.476 ↓	0.617 ↓	0.811 ↑	0.429 ↑	1.000 ↓	0.038 ↓	0.717 ↓	**3.8E-08** ↑	**0.001** ↑	**3.8E-08** ↑
22	1.000 ↓	1.000 ↓	0.850 ↑	1.000 ↓	0.160 ↓	0.050 ↓	0.198 ↑	1.000 ↓	0.603 ↑	0.354 ↑	1.000 ↓	0.336 ↑	0.471 ↑	0.042 ↑	0.471 ↑
23	1.000 ↓	0.109 ↓	0.274 ↓	0.458 ↓	0.891 ↓	1.000 ↓	0.059 ↑	1.000 ↓	0.833 ↑	0.855 ↑	1.000 ↓	0.849 ↑	0.509 ↓	1.000 ↓	0.509 ↑
24	0.440 ↑	0.859 ↑	0.353 ↓	0.185 ↓	0.290 ↑	0.232 ↑	0.603 ↑	0.168 ↑	0.032 ↑	0.201 ↓	0.083 ↓	0.423 ↓	0.058 ↓	0.862 ↓	0.058 ↓
25	0.431 ↓	0.597 ↓	0.227 ↓	0.014 ↓	0.567 ↑	0.080 ↓	0.662 ↑	0.262 ↑	0.327 ↑	0.848 ↓	0.084 ↓	0.020 ↑	0.771 ↓	0.141 ↓	0.771 ↑
26	0.790 ↓	0.475 ↑	0.090 ↓	0.885 ↓	0.064 ↓	0.862 ↑	0.687 ↑	0.520 ↓	0.835 ↓	1.000 ↓	0.843 ↑	0.137 ↑	0.189 ↓	0.761 ↑	0.189 ↑
27	0.432 ↑	0.078 ↑	**0.003** ↑	0.869 ↑	0.115 ↑	0.586 ↓	0.171 ↓	0.514 ↑	0.247 ↓	0.108 ↓	0.635 ↑	0.853 ↓	0.112 ↑	**1.9E-04** ↑	0.112 ↑
28	0.034 ↓	0.298 ↓	0.705 ↓	0.286 ↓	0.473 ↑	1.000 ↓	1.000 ↓	0.230 ↓	1.000 ↓	0.710 ↑	0.813 ↓	0.227 ↓	0.028 ↓	0.031 ↑	0.028 ↑
29	0.789 ↓	0.489 ↓	0.858 ↑	0.541 ↑	0.676 ↑	0.599 ↓	1.000 ↓	0.667 ↑	1.000 ↓	0.280 ↑	1.000 ↓	0.853 ↓	0.432 ↓	0.534 ↓	0.432 ↓
30	0.364 ↑	0.860 ↑	0.365 ↑	0.739 ↑	0.429 ↑	0.081 ↓	0.305 ↓	0.659 ↓	1.000 ↓	0.109 ↓	0.817 ↑	0.713 ↑	0.197 ↑	**0.009** ↑	0.197 ↑

A nucleotide ↑: frequency is increased in HEG compared to all genes.↓: frequency is decreased in HEG compared to all genes

nucleotide position	organism														
	ecoli	bsubt	hpylo	hinfl	mtube	tpall	rprow	mgeni	bburg	afulg	mjann	phori	scere	spomb	pfalc
4	**0.009** ↓	0.129 ↓	0.779 ↑	**0.010** ↓	0.430 ↓	0.441 ↑	0.304 ↓	0.427 ↓	0.017 ↓	0.267 ↓	0.020 ↓	0.575 ↓	**2.3E-06** ↓	0.044 ↓	**0.002** ↓
5	0.057 ↓	**0.003** ↓	0.120 ↓	**0.008** ↓	0.478 ↓	0.751 ↑	**0.006** ↓	0.212 ↓	**0.006** ↓	0.197 ↓	0.016 ↓	0.302 ↓	**4.7E-04** ↓	**3.8E-04** ↓	**4.7E-07** ↓
6	0.110 ↑	0.199 ↑	**0.008** ↑	0.540 ↑	0.100 ↑	0.151 ↑	0.304 ↑	0.350 ↑	0.227 ↑	0.351 ↓	0.013 ↑	0.879 ↓	**0.004** ↓	0.099 ↑	**0.002** ↑
7	0.422 ↑	0.782 ↓	0.069 ↑	0.625 ↓	**0.004** ↑	0.208 ↑	0.883 ↑	0.280 ↑	0.656 ↑	0.385 ↑	0.786 ↑	0.888 ↑	0.478 ↑	1.000 ↓	0.240 ↑
8	0.643 ↑	0.783 ↑	0.783 ↑	0.046 ↓	0.104 ↑	0.186 ↑	0.077 ↓	0.639 ↓	0.457 ↓	0.040 ↑	0.671 ↓	0.026 ↑	0.906 ↑	0.782 ↓	0.381 ↑
9	0.711 ↑	**0.002** ↑	0.569 ↑	0.019 ↑	0.721 ↑	0.853 ↑	0.379 ↑	0.115 ↑	0.765 ↑	0.210 ↑	0.326 ↑	0.761 ↑	0.185 ↑	0.048 ↓	0.364 ↑
10	**7.8E-05** ↑	0.258 ↑	0.016 ↑	0.062 ↑	0.070 ↑	0.137 ↑	0.106 ↑	0.640 ↑	0.139 ↑	0.042 ↑	0.684 ↓	0.158 ↑	0.344 ↓	0.268 ↓	0.553 ↑
11	0.459 ↑	0.669 ↓	0.774 ↑	0.523 ↑	0.205 ↑	1.000 ↓	0.051 ↑	0.117 ↑	0.224 ↑	0.092 ↑	0.885 ↓	0.209 ↓	0.018 ↓	0.494 ↑	0.306 ↑
12	0.541 ↑	0.015 ↑	0.305 ↑	0.536 ↑	0.464 ↑	0.584 ↑	0.380 ↑	0.208 ↑	1.000 ↑	0.145 ↓	0.396 ↑	0.210 ↓	0.076 ↓	0.492 ↓	0.023 ↑
13	**0.007** ↑	0.477 ↑	0.047 ↑	0.045 ↑	**1.4E-04** ↑	**0.001** ↑	0.558 ↑	0.755 ↓	**0.002** ↑	0.464 ↑	0.497 ↑	0.472 ↓	0.293 ↑	0.013 ↑	0.106 ↑
14	0.013 ↑	0.476 ↓	0.564 ↑	0.606 ↓	0.033 ↑	0.862 ↑	0.652 ↑	0.752 ↑	0.446 ↑	0.174 ↑	0.400 ↑	0.174 ↑	0.243 ↑	0.596 ↓	0.240 ↓
15	0.055 ↑	0.775 ↓	0.466 ↑	0.703 ↓	0.199 ↓	1.000 ↓	0.766 ↑	0.211 ↑	0.880 ↑	1.000 ↑	0.488 ↑	0.646 ↑	0.813 ↑	**0.007** ↓	0.175 ↑
16	0.548 ↓	0.085 ↓	**0.009** ↓	0.701 ↓	0.062 ↑	0.398 ↑	0.769 ↓	1.000 ↑	0.653 ↓	0.661 ↓	0.276 ↑	0.080 ↓	0.724 ↓	0.891 ↓	0.659 ↑
17	1.000 ↓	0.242 ↑	0.382 ↑	0.435 ↑	0.019 ↑	0.020 ↑	0.292 ↓	0.530 ↓	0.044 ↑	0.878 ↓	0.670 ↑	0.219 ↑	1.000 ↓	0.580 ↓	0.025 ↓
18	1.000 ↑	0.666 ↓	0.765 ↓	0.613 ↑	0.177 ↓	0.356 ↑	0.303 ↑	0.874 ↓	0.170 ↑	0.737 ↓	0.784 ↑	0.267 ↓	**0.003** ↓	0.671 ↓	0.066 ↑
19	0.902 ↓	0.666 ↓	0.669 ↑	0.699 ↑	0.024 ↑	0.169 ↑	0.882 ↓	0.874 ↓	0.657 ↑	0.369 ↓	0.412 ↓	0.772 ↑	0.639 ↓	0.781 ↑	0.769 ↓
20	0.614 ↑	0.104 ↑	0.772 ↑	0.364 ↓	0.161 ↑	0.195 ↑	0.881 ↑	0.869 ↑	0.213 ↑	1.000 ↓	1.000 ↑	0.164 ↑	0.074 ↑	0.166 ↑	0.055 ↑
21	0.353 ↓	0.242 ↑	0.768 ↑	0.702 ↑	1.000 ↓	0.448 ↑	0.769 ↓	0.411 ↑	0.762 ↓	0.869 ↓	0.074 ↑	0.650 ↓	0.464 ↓	**0.003** ↓	0.763 ↑
22	0.805 ↓	0.384 ↑	0.305 ↑	0.794 ↓	0.250 ↑	0.025 ↑	0.137 ↓	0.588 ↑	0.551 ↑	0.052 ↑	0.338 ↑	0.663 ↑	0.724 ↓	0.676 ↓	0.771 ↓
23	0.899 ↑	0.183 ↑	0.139 ↑	0.794 ↑	0.083 ↑	0.055 ↑	0.532 ↑	0.617 ↑	**0.002** ↑	0.442 ↓	0.671 ↑	0.087 ↑	0.406 ↑	0.680 ↓	0.378 ↓
24	1.000 ↓	0.459 ↓	0.378 ↑	0.157 ↑	0.841 ↓	0.244 ↓	0.882 ↑	0.424 ↓	0.362 ↓	0.317 ↑	0.028 ↑	0.218 ↓	0.118 ↑	0.666 ↓	0.453 ↑
25	0.262 ↑	0.657 ↓	0.242 ↑	0.118 ↑	0.046 ↑	0.048 ↑	0.555 ↓	0.532 ↑	0.880 ↑	0.079 ↑	0.051 ↓	1.000 ↑	0.722 ↑	0.050 ↑	**2.0E-04** ↑
26	0.797 ↑	1.000 ↓	1.000 ↓	0.894 ↓	0.231 ↑	0.861 ↓	0.645 ↑	0.609 ↓	0.527 ↓	0.132 ↑	0.888 ↓	0.878 ↑	0.906 ↑	0.264 ↑	1.000 ↓
27	0.587 ↑	0.372 ↑	0.878 ↓	0.307 ↑	0.545 ↓	0.700 ↓	0.765 ↓	0.739 ↓	0.449 ↑	0.620 ↓	0.098 ↑	1.000 ↓	0.460 ↑	0.029 ↓	0.881 ↓
28	0.022 ↑	0.233 ↑	0.039 ↑	0.041 ↓	0.524 ↑	0.167 ↑	0.657 ↓	0.622 ↑	1.000 ↓	0.224 ↑	0.491 ↑	0.462 ↑	0.031 ↑	1.000 ↓	0.458 ↑
29	0.157 ↑	0.177 ↑	0.762 ↑	0.427 ↑	0.147 ↑	1.000 ↓	1.000 ↑	1.000 ↑	0.424 ↓	0.366 ↑	0.478 ↑	0.879 ↓	0.630 ↓	0.780 ↑	1.000 ↓
30	0.489 ↑	0.033 ↑	1.000 ↑	0.406 ↑	0.404 ↓	0.852 ↑	0.136 ↑	0.630 ↓	0.880 ↓	0.866 ↑	0.681 ↑	0.649 ↑	0.038 ↓	0.059 ↓	0.879 ↓

G nucleotide ↑: frequency is increased in HEG compared to all genes. ↓: frequency is decreased in HEG compared to all genes

nucleotide position	organism														
	ecoli	bsubt	hpylo	hinfl	mtube	tpall	rprow	mgeni	bburg	afulg	mjann	phori	scere	spomb	pfalc
4	**0.005** ↑	0.049 ↑	0.024 ↑	**0.001** ↑	**0.008** ↑	**0.005** ↑	**2.2E-04** ↑	0.064 ↑	**4.6E-04** ↑	**0.004** ↑	**0.006** ↑	0.541 ↑	**1.7E-04** ↑	**8.1E-04** ↑	**0.001** ↑
5	0.366 ↓	0.553 ↑	1.000 ↓	1.000 ↓	0.118 ↓	0.255 ↓	**0.003** ↑	0.113 ↑	0.014 ↑	0.287 ↓	0.134 ↑	0.734 ↑	0.875 ↓	0.167 ↑	0.224 ↑
6	**7.8E-05** ↓	0.107 ↓	0.673 ↓	0.013 ↓	0.673 ↓	0.240 ↑	0.285 ↑	0.588 ↑	0.386 ↓	0.106 ↑	0.074 ↓	0.553 ↓	0.068 ↓	0.061 ↓	0.674 ↓
7	0.142 ↑	0.064 ↑	0.256 ↓	0.449 ↑	0.111 ↓	1.000 ↓	0.157 ↑	0.836 ↑	1.000 ↓	0.471 ↓	0.435 ↑	0.879 ↓	0.375 ↑	0.886 ↓	0.863 ↓
8	1.000 ↓	0.676 ↓	0.497 ↑	0.836 ↑	0.628 ↓	1.000 ↓	1.000 ↓	0.155 ↑	0.382 ↑	0.144 ↓	0.096 ↑	0.373 ↓	0.030 ↑	0.698 ↑	0.380 ↑
9	0.893 ↓	0.033 ↓	0.847 ↓	0.515 ↓	1.000 ↑	0.640 ↑	0.637 ↑	0.806 ↓	0.393 ↑	0.031 ↑	1.000 ↓	0.544 ↑	0.100 ↓	1.000 ↓	1.000 ↓
10	0.781 ↓	0.031 ↑	0.866 ↑	0.342 ↑	0.274 ↓	0.521 ↓	0.834 ↓	1.000 ↓	0.857 ↑	0.558 ↑	0.530 ↑	0.209 ↓	**0.008** ↑	0.106 ↑	1.000 ↓
11	0.267 ↓	0.405 ↑	1.000 ↓	0.425 ↑	0.337 ↓	0.722 ↓	0.568 ↑	0.608 ↓	0.385 ↓	0.847 ↓	0.034 ↑	0.838 ↓	1.000 ↓	0.688 ↑	1.000 ↓
12	**1.7E-05** ↓	0.026 ↓	0.839 ↑	0.084 ↓	0.055 ↓	1.000 ↓	0.517 ↓	0.141 ↑	0.395 ↑	0.133 ↑	1.000 ↓	0.039 ↑	0.032 ↓	0.586 ↓	0.814 ↓
13	0.686 ↓	0.330 ↑	0.512 ↑	0.648 ↑	0.689 ↑	0.513 ↓	0.695 ↑	0.692 ↑	0.437 ↓	1.000 ↓	0.454 ↑	0.229 ↑	0.298 ↑	1.000 ↓	0.852 ↑
14	0.049 ↓	1.000 ↓	0.510 ↓	1.000 ↓	0.022 ↓	0.164 ↓	0.763 ↓	0.616 ↑	0.635 ↑	**0.004** ↑	0.273 ↑	0.335 ↑	0.021 ↓	0.057 ↓	0.653 ↓
15	0.392 ↓	0.108 ↓	0.111 ↑	0.027 ↓	0.493 ↑	1.000 ↓	0.169 ↓	1.000 ↓	0.658 ↑	0.132 ↑	0.738 ↓	0.755 ↓	0.460 ↓	1.000 ↓	0.488 ↑
16	0.016 ↑	0.083 ↑	**0.005** ↑	0.136 ↑	0.231 ↓	1.000 ↓	0.688 ↑	0.236 ↑	0.577 ↑	0.012 ↑	0.536 ↓	0.228 ↑	0.153 ↑	0.023 ↑	0.583 ↑
17	0.205 ↑	0.535 ↑	1.000 ↓	0.699 ↑	0.052 ↓	0.736 ↑	0.302 ↑	1.000 ↓	0.641 ↑	1.000 ↑	1.000 ↓	1.000 ↓	0.270 ↓	0.729 ↑	0.827 ↑
18	0.586 ↓	0.729 ↑	0.340 ↑	0.679 ↓	0.139 ↑	0.324 ↓	0.791 ↓	0.808 ↓	1.000 ↑	0.442 ↑	0.581 ↓	0.267 ↓	0.051 ↓	0.322 ↓	0.269 ↓
19	0.800 ↑	0.876 ↓	0.748 ↑	0.251 ↑	0.510 ↓	0.633 ↓	0.583 ↑	0.307 ↓	0.581 ↑	0.561 ↑	0.293 ↑	0.441 ↑	0.183 ↑	0.655 ↑	0.031 ↑
20	0.647 ↓	0.310 ↑	0.198 ↓	0.173 ↑	0.031 ↓	0.382 ↑	0.081 ↑	0.340 ↓	0.147 ↑	0.852 ↑	1.000 ↓	1.000 ↑	0.026 ↓	0.016 ↓	0.066 ↑
21	0.408 ↑	0.377 ↓	0.849 ↑	1.000 ↓	1.000 ↑	0.627 ↑	0.571 ↓	1.000 ↓	0.521 ↑	0.875 ↓	0.711 ↑	1.000 ↓	0.058 ↓	0.849 ↓	0.269 ↓
22	0.129 ↑	0.878 ↓	0.267 ↑	0.481 ↑	0.693 ↑	0.274 ↑	**0.002** ↑	0.857 ↓	0.106 ↑	0.192 ↓	1.000 ↓	0.548 ↓	0.155 ↑	0.375 ↑	0.360 ↑
23	0.298 ↓	0.151 ↑	0.401 ↑	0.706 ↑	0.199 ↓	0.476 ↓	0.358 ↑	0.390 ↑	0.320 ↑	0.719 ↓	0.078 ↑	0.847 ↓	0.015 ↑	0.071 ↑	0.654 ↑
24	0.785 ↓	0.610 ↓	0.454 ↑	0.700 ↑	0.140 ↓	0.627 ↓	0.420 ↓	0.387 ↑	1.000 ↓	0.645 ↑	0.854 ↓	1.000 ↓	0.058 ↓	0.734 ↓	0.479 ↓
25	0.052 ↑	0.224 ↑	0.432 ↑	0.018 ↑	0.361 ↓	0.222 ↑	0.045 ↑	0.446 ↓	0.472 ↑	0.884 ↓	0.017 ↑	0.366 ↓	0.441 ↑	0.306 ↑	0.716 ↓
26	0.557 ↑	1.000 ↑	**0.005** ↑	0.479 ↑	1.000 ↓	0.295 ↑	0.442 ↑	0.822 ↑	0.169 ↑	1.000 ↓	0.340 ↑	0.053 ↑	1.000 ↓	0.857 ↓	0.241 ↑
27	0.793 ↓	0.016 ↓	0.468 ↓	1.000 ↓	0.347 ↓	0.639 ↑	0.109 ↑	0.800 ↑	0.162 ↑	**0.003** ↑	0.144 ↓	0.179 ↑	0.153 ↓	0.311 ↑	0.115 ↓
28	0.226 ↑	0.370 ↑	0.875 ↓	**0.002** ↑	0.512 ↓	0.753 ↓	1.000 ↑	0.227 ↑	0.023 ↑	0.885 ↓	0.764 ↑	0.182 ↑	0.306 ↑	0.139 ↑	0.026 ↑
29	0.662 ↑	0.076 ↑	0.432 ↑	0.509 ↑	0.523 ↓	0.732 ↑	0.169 ↓	0.645 ↑	0.024 ↑	0.594 ↑	0.128 ↑	0.138 ↑	0.013 ↑	0.013 ↑	**0.010** ↑
30	0.248 ↓	**0.007** ↓	0.145 ↑	0.013 ↓	0.790 ↑	0.635 ↑	0.868 ↓	1.000 ↓	0.824 ↓	0.286 ↑	1.000 ↓	0.309 ↓	0.666 ↓	0.095 ↓	0.530 ↑
