# Supplementary material for: Highly expressed proteins have an increased frequency of alanine in the second amino acid position
Source: BMC Genomics. 2006 Feb 16;7:28. doi: 10.1186/1471-2164-7-28 (PMC1397820; doi:10.1186/1471-2164-7-28)
Supplement: Additional File 3 — Justifying the method of using orthologues. [file 1471-2164-7-28-S3.pdf]

Additional file 3.

Although the method of using orthologues has been successfully used in previous studies [41, 42] there is still concern that different genes might be highly expressed in different organisms.

The codon usage of a particular organism can be analysed by correspondence analysis. We have done this analysis for the organisms used in the current study (Fig. A3.1. below). We observed that in many organisms the HEG that we have defined by the method of orthologues cluster together (shown in red). In addition, the HEG usually cluster together with the genes for ribosomal proteins (shown in green) known to have high expression level. We observed that in organisms where the HEG are well separated from other genes, there are additional genes with a similar codon usage as in the HEG defined by the method of orthologues. In case the overrepresentation of alanine is specific for highly expressed genes then this pattern should be observed also in these additional genes (Table A3.2. below). We have performed this analysis in *B.subtilis, H.influenzae, M.jannaschii* and *S.pombe* (Table A3.1. below). The results show that in *B.subtilis* and *S.pombe* the p-value for alanine overrepresentation in HEG is even decreased. In *M.jannaschii* the p-value has increased but is still significant. In *H.influenzae* the p-value has increased to 0.03 (alanine overrepresentation is not considered significant). We conclude that in most organisms the alanine overrepresentation is still present in additional highly expressed genes.

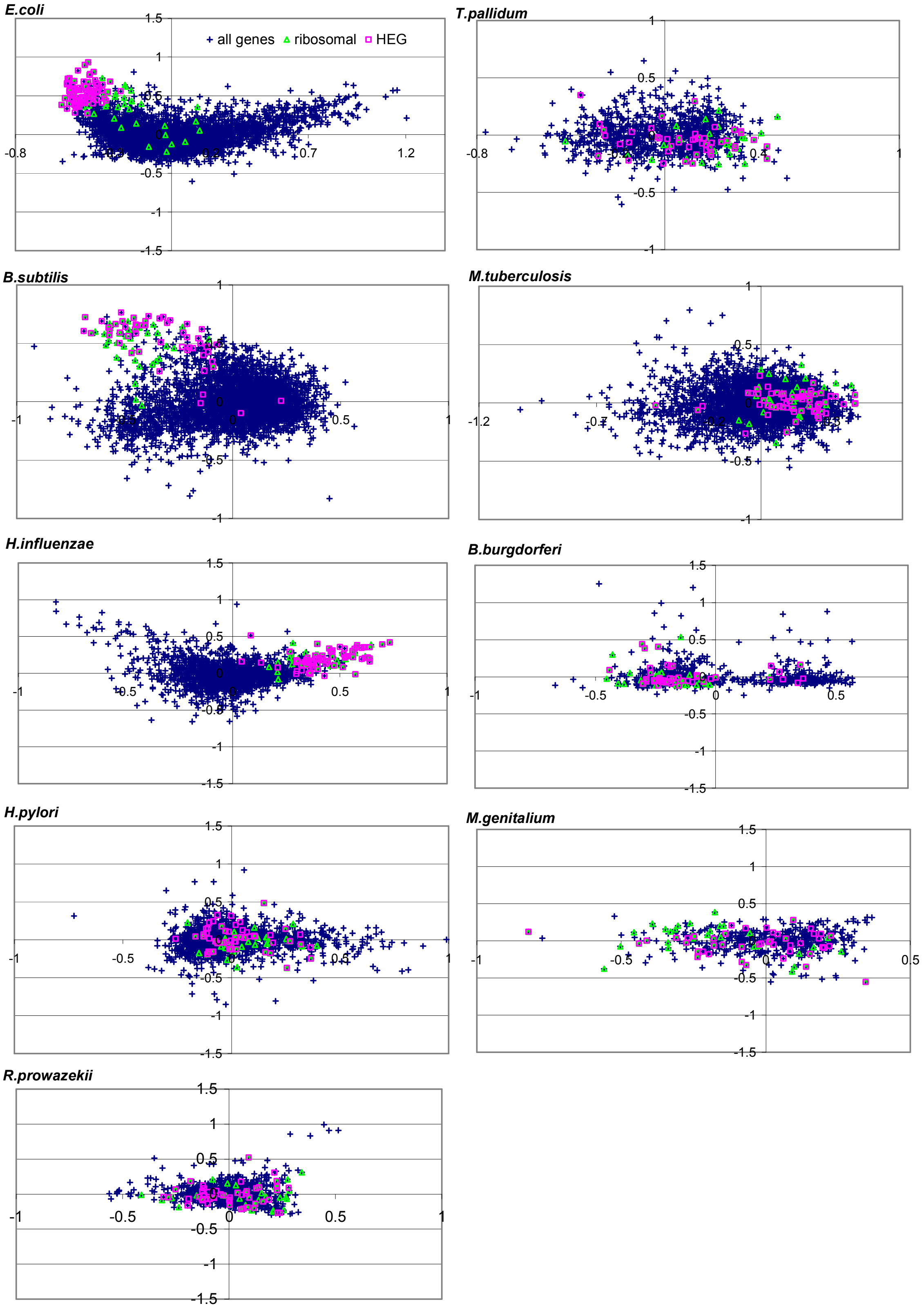

Figure A3.1. The correspondence analysis of relative synonymous codon usage.

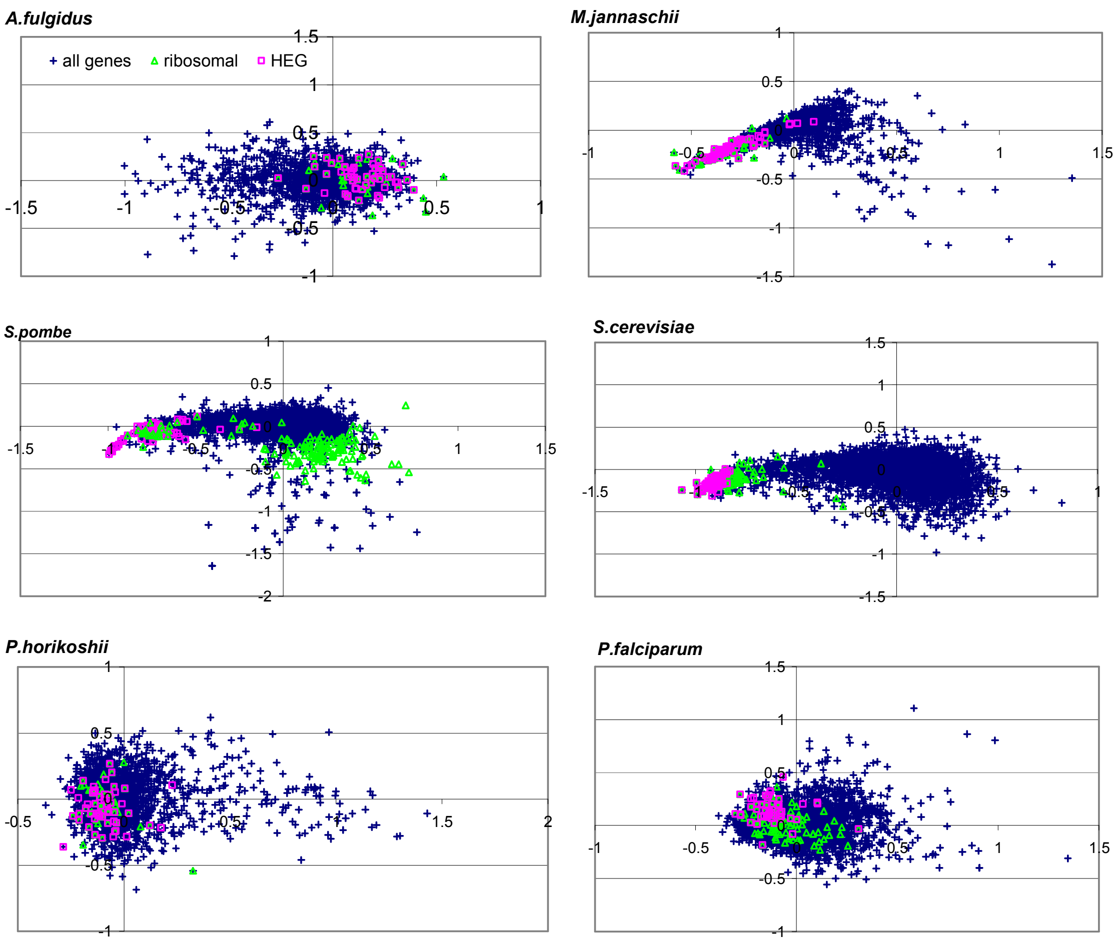

Figure A3.1 (continued). The correspondence analysis of relative synonymous codon usage.

Table A3.1. Preference for amino acids at the beginning of additional highly expressed proteins compared to all proteins datasets. (H_0: there is no difference of amino acid frequencies between all proteins and highly expressed proteins).

organism	amino acid position											
	2			3			4			5		
	amino acid	P-value	%HEG/ %all	amino acid	P-value	%HEG/ %all	amino acid	P-value	%HEG/ %all	amino acid	P-value	%HEG/ %all
B.subtilis	**Ala**	2.6E-08	24/7		-			-			-	
H.influenzae		-			-			-			-	
M.jannaschii	**Ala**	2.0E-05	14/4		-			-			-	
S.pombe	**Ala**	4.8E-06	25/10	Ala	0.008	12/5		-		Lys	0.004	17/8
	Asn	0.002	0/6									

Table A3.2. List of the additional highly expressed genes.

E.coli		*B.subtilis*		*H.influenzae*		*M.jannaschii*		*S.pombe*	
accB	nusG	abrB	sspJ	HI0084	HI0796	MJ0032	MJ0868	SPAC1002.13c	SPBC19G7.03c
accC	pckA	aprE	tatAY	HI0110	HI0800	MJ0040	MJ0926	SPAC1002.17c	SPBC1E8.05
aceF	pepD	atpE	trxA	HI0119	HI0801	MJ0049	MJ0932	SPAC1002.19	SPBC215.05
acnB	pgi	atpF	yaaD	HI0131	HI0803	MJ0081	MJ0959	SPAC1006.07	SPBC21C3.13
allB	pgm	clpX	ybfE	HI0142	HI0808	MJ0083	MJ0960	SPAC1071.08	SPBC28F2.03
ansB	pheS	comX	yddQ	HI0146	HI0809	MJ0097	MJ0990	SPAC1071.10c	SPBC29A10.08
argS	pheT	cspD	ydfP	HI0155	HI0824	MJ0098	MJ1035	SPAC11D3.01c	SPBC29B5.03c
artI	proS	dltC	yetH	HI0158	HI0834	MJ0112	MJ1045	SPAC11G7.04	SPBC2F12.04
asnS	prsA	fbaA	yfhD	HI0170	HI0835	MJ0113	MJ1090	SPAC13G6.02c	SPBC2G5.05
aspA	pstS	flgE	yhbJ	HI0171	HI0843	MJ0168	MJ1091	SPAC144.11	SPBC32H8.12c
aspS	ptsG	fliL	yhcN	HI0201	HI0847	MJ0199	MJ1099	SPAC1687.06c	SPBC336.10c
atpE	pyrG	frr	yhfD	HI0221	HI0880	MJ0212	MJ1118	SPAC1783.08c	SPBC354.12
b2339	recA	fusA	yjbD	HI0221.1	HI0916	MJ0216	MJ1142	SPAC1805.11c	SPBC365.03c
cadA	rho	glnA	yjcS	HI0222	HI0924	MJ0217	MJ1148	SPAC1805.13	SPBC3D6.02
cchA	rplE	groES	yjdB	HI0229	HI0927	MJ0220	MJ1153	SPAC1834.03c	SPBC3D6.15
crp	rplP	guaB	yjlD	HI0241	HI0928	MJ0221	MJ1156	SPAC1834.04	SPBC428.05c
crr	rplR	hag	ykuU	HI0246	HI0937	MJ0222	MJ1167	SPAC18G6.14c	SPBC530.10c
cydA	rplV	infA	ykwD	HI0250	HI0951	MJ0226	MJ1168	SPAC19G12.06c	SPBC56F2.02
cydB	rplX	lysS	ymzB	HI0315	HI0968	MJ0242	MJ1169	SPAC19G12.08	SPBC660.16
dapD	rpmC	lytA	yneF	HI0317	HI0971	MJ0268	MJ1171	SPAC1A6.04c	SPBC685.06
deaD	rpmF	murAA	ynzC	HI0323	HI0972	MJ0269	MJ1186	SPAC1F12.02c	SPBC776.01
deoB	rpoZ	pdhA	yocJ	HI0325	HI0980	MJ0280	MJ1192.1	SPAC1F7.13c	SPBC800.04c
dsbA	rpsE	pdhB	yodC	HI0348	HI0989	MJ0285	MJ1199	SPAC1F8.07c	SPBC839.05c
eda	rpsH	pdhC	yonB	HI0362	HI1034	MJ0299	MJ1201	SPAC23A1.11	SPBC839.13c
fabA	rpsJ	ppiB	yonC	HI0370	HI1050	MJ0307	MJ1202	SPAC23C11.05	SPBC8D2.03c
fabI	secA	pstS	yonK	HI0371	HI1053	MJ0308	MJ1203	SPAC23H4.06	SPBC8D2.04
frdA	secB	ptsH	yopL	HI0377	HI1061	MJ0318	MJ1258	SPAC24C9.12c	SPBC8D2.18c
frr	ssb	rplE	yoqO	HI0411	HI1062	MJ0327	MJ1271	SPAC24H6.07	SPBP8B7.03c
ftsZ	sucB	rplF	yosA	HI0445	HI1069	MJ0362	MJ1273	SPAC25B8.12c	SPBP8B7.06
glmS	sucC	rplJ	yppF	HI0483	HI1075	MJ0386	MJ1277	SPAC26A3.04	SPCC1020.06c
glnH	talB	rplN	yqbN	HI0484	HI1076	MJ0394	MJ1325	SPAC26H5.10c	SPCC1223.09
gltA	tpx	rplP	yqdA	HI0494	HI1124	MJ0396	MJ1346	SPAC27E2.11c	SPCC1259.01c
gltX	tyrS	rplQ	yqeY	HI0495	HI1125	MJ0401	MJ1391	SPAC29A4.02c	SPCC1281.06c
gnd	udp	rplR	yrbF	HI0515	HI1172	MJ0402	MJ1419	SPAC31G5.03	SPCC1322.10
gntT	uxaC	rplS	yrhC	HI0519	HI1193	MJ0404	MJ1455	SPAC31G5.17c	SPCC1322.11
gpmA	valS	rplU	yrkE	HI0534	HI1204	MJ0458.1	MJ1509	SPAC343.12	SPCC1393.03
gpt	yajC	rplV	yuaB	HI0542	HI1210	MJ0459	MJ1511	SPAC3A12.10	SPCC1672.02c
groS	ydgH	rplW	yuaJ	HI0553	HI1211	MJ0466	MJ1534	SPAC3H5.05c	SPCC1682.14
guaA	ydgR	rplX	yugI	HI0557	HI1232	MJ0467	MJ1573	SPAC3H5.07	SPCC1739.13
gyrA	ydhD	rpmB	yuiA	HI0572	HI1252	MJ0468	MJ1575	SPAC3H5.12c	SPCC191.07
hflB	yeeF	rpmC	yusA	HI0580	HI1264	MJ0473	MJ1601	SPAC4F10.20	SPCC24B10.09
hflC	ygeY	rpmD	yvaB	HI0585	HI1284	MJ0476	MJ1656	SPAC4F8.07c	SPCC31H12.04c
hslU	yggV	rpmGA	yvcE	HI0591	HI1302	MJ0477	MJ1670	SPAC521.05	SPCC330.06c
htpG	yibO	rpmH	yvzB	HI0620	HI1318	MJ0498		SPAC57A7.04c	SPCC330.14c
hupB	yidC	rpmI	ywkA	HI0640	HI1333	MJ0546		SPAC664.04c	SPCC364.03
icdA	yjgF	rpoA	yydF	HI0646	HI1344	MJ0595		SPAC664.05	SPCC417.08
ilvC	yjiY	rpoB		HI0682	HI1354	MJ0608		SPAC6G10.11c	SPCC553.10
infB		rpsD		HI0686	HI1363	MJ0648		SPAC890.08	SPCC576.03c
iscS		rpsE		HI0693	HI1367	MJ0655		SPAC8C9.08	SPCC576.11
iscU		rpsF		HI0736	HI1390	MJ0657		SPAC9.09	SPCC613.06
katG		rpsG		HI0738	HI1391	MJ0659		SPAC926.04c	SPCC622.08c
leuS		rpsH		HI0740	HI1396	MJ0673		SPAC959.08	SPCC622.09
lysS		rpsJ		HI0746	HI1398	MJ0691		SPACUNK4.17	SPCC622.12c
malE		rpsK		HI0757	HI1434.1	MJ0707		SPAPB24D3.07c	SPCC622.18
manZ		rpsM		HI0776	HI1522.1	MJ0728		SPAPB8E5.06c	SPCC736.15
menB		rpsR		HI0782	HI1532	MJ0746		SPBC106.18	
metK		rpsS		HI0784	HI1545	MJ0784		SPBC1105.12	
metQ		rpsT		HI0788	HI1573	MJ0800		SPBC14F5.05c	
mglB		rpsU		HI0789	HI1609	MJ0822		SPBC1604.05	
mipA		secE		HI0790	HI1633	MJ0825		SPBC1685.09	
mreB		sodA		HI0791	HI1634	MJ0842		SPBC1685.13	
mtlA		spoIIQ		HI0792	HI1659	MJ0845		SPBC16H5.02	
nagE		sspA		HI0793	HI1693	MJ0846		SPBC17G9.10	
nupC		sspB		HI0794	HI1702	MJ0854		SPBC18E5.06	
nusA		sspC		HI0795	HI1711	MJ0863		SPBC18H10.12c	
					HI1734			SPBC19F8.08	
Total:	112	Total:	111	Total:	131	Total:	108	Total:	121
