# Supplementary material for: Highly expressed proteins have an increased frequency of alanine in the second amino acid position
Source: BMC Genomics. 2006 Feb 16;7:28. doi: 10.1186/1471-2164-7-28 (PMC1397820; doi:10.1186/1471-2164-7-28)
Supplement: Additional File 4 — Justifying the selection of organism. [file 1471-2164-7-28-S4.pdf]

Additional file 4.

When selecting the organisms for the current study we attempted to include genomes with different properties (size, GC content). Still, the question remains, how general is our observation of overrepresentation of alanine in the second position of HEG? To answer this question we chose from sequenced genomes by random number generator three additional bacterial (*Mycobacterium avium paratuberculosis, Synechococcus elongatus, Rickettsia conorii*), one archaeal (*Haloarcula marismortui*) and one unicellular eukaryotic genome (*Cryptococcus neoformans*). We found the HEG by the method of orthologues (Table A4.2. below). The significant overrepresentation of alanine in the second position of HEG was observed in four out of the five genomes (Table A.4.1. below). Also, the frequency of alanine was 2.3 times increased in *R.conorii*, although the statistical significance was low (P=0.043). These results suggest that the trend is present in most unicellular organisms.

Table A4.1. Preference for amino acids at the beginning of highly expressed proteins compared to all proteins datasets. (H_0: there is no difference of amino acid frequencies between all proteins and highly expressed proteins).

organism	amino acid position											
	2			3			4			5		
	amino acid	P-value	%HEG/ %all	amino acid	P-value	%HEG/ %all	amino acid	P-value	%HEG/ %all	amino acid	P-value	%HEG/ %all
S.elongatus	**Ala**	2.4E-04	34/14	Lys	1.5E-05	22/5	Lys	3.9E-04	14/3		-	
M.avium paratuberculosis	**Ala**	8.6E-10	46/14	Lys	1.3E-04	14/3	Lys	4.3E-05	14/3	Lys	2.3E-04	13/3
R.conorii		**-**		Thr	9.0E-04	17/4		-			-	
H.marismortui	**Ala**	6.8E-06	31/11		-		Glu	0.004	18/7		-	
C.neoformans	Gly<br>**Ala**	0.001<br>0.003	14/4<br>28/14	Lys	0.002	14/4	Val<br>Lys	0.005<br>0.009	13/4<br>13/5	Lys	5.2E-04	17/5

Table A4.2. List of orthologues.

E.coli **(80)**	*H.marismortui (33+34)*	*M.avium paratuberculosis (63)*	*R.conorii (53)*	*S.elongatus (59)*
lpp				
rplL		+	+	+
eno	+	+		+
gapA	+	+		+
ompC				
tufA	+			
cspA	+	+		
ahpC	+	+	+	+
tufB		+	+	+
mopA(groL)	+	+	+	+
ompA				
rpsI		+	+	+
rpsA		+		+
pflB				
rpsB		+	+	+
fba				+
tsf		+	+	+
rplA	+	+	+	+
rplY		+	+	
fusA	+	+	+	+
rpmA		+	+	+
tpiA		+		+
rpmE		+		+
ompX				
tig		+	+	+
pgk	+	+		+
rpsC	+	+		+
rpmG		+	+	
rpsU				+
rpmH		+	+	+
rplI		+	+	+
dnaK	+	+	+	+
sodA	+	+	+	+
rpmI		+		+
yaiU				
rplB	+	+		+
rplC	+	+	+	+
yfiD				
rpsQ	+	+	+	+
pykF	+	+		+
rplK	+	+	+	+
rplO		+	+	+
rplD		+	+	+
rpoC	+	+	+	+
rpsO		+	+	+
cspC	+		+	
efp		+	+	+
slyD	+			
pal			+	
trpL				
tktA		+		+
rplT		+	+	+
rpsF		+	+	+
aceE		+		
atpA		+	+	+
ppiB	+	+		+
acpP		+	+	+
pta	+	+	+	
rpsR		+	+	+
glyA	+	+	+	+
hupA		+	+	+
rplM	+	+	+	+
rpsT		+	+	+
ompF				
pfkA		+		+
rpsL	+	+	+	+
ackA		+	+	+
rpsP		+	+	+
ppa	+	+	+	+
deoD	+			
lpdA	+	+	+	+
adhE	+	+	+	
rplW	+	+	+	+
atpD	+	+	+	+
adk	+	+	+	+
ybaB		+	+	+
bipA		+	+	+
uxuA				
ndk	+	+	+	+
yebC		+	+	+

Table 4A.2 (continued). List of orthologues

***S.cerevisiae* (80)**	*C.neoformans (64)*	*H.marismortui (33+34)*
TDH3		
RPL39	+	+
TDH2		
PDC1	+	+
CDC19	++	
ENO2		
TEF2	+	
RPL43A	+	+
ENO1	+	
TEF1		
CCW12		
FBA1	+	
RPL30	+	
RPL22A	+	
TDH1	+	
RPL8B		
RPS6B	+	+
RPS12	+	
RPL8A	+	+
RPS10A	+	
RPS5	+	+
RPS6A		
RPL1B	+	
RPL3	+	
RPL5	+	+
RPS20	+	+
RPL10	+	+
RPL1A		
SSB1		
RPL32	+	+
TPI1	+	
RPS24A	+	+
PGK1	++	
HYP2	+	+
RPL4B	+	+
RPS22A	+	+
RPL18A	+	
GPM1		+
RPS31	+	
ADH1	+	+
RPS9B	+	+
RPS17A	+	+
RPL17A	+	+
RPS14A	+	+
RPP2A	+	+
EFT1	+	
RPL4A		
SSA2	++	
ILV5	+	+
RPS10B		
RPS2	+	
EFT2		
RPS3	+	
RPL36B	+	
RPL35A	+	
RPL42B		
RPP0	+	+
RPS19A	+	+
RPS23B	+	+
RPL15A	+	+
RPS26A	+	
RPL34B	+	
RPL11A	+	+
YEF3	+	+
RPL9B	+	+
ASC1	+	
RPS13	+	+
RPS18A	+	+
RPL38		
RPL2A	+	
SSB2	+	
RPL9A		
RPS1B	+	+
RPL42A		
RPS15	+	+
RPL12B	+	
CWP2		
RPS16B	+	+
RPL2B		
EFB1	+	
